# Rasch Analysis of the Norwegian Version of the Occupational Balance Questionnaire in a Sample of Occupational Therapy Students

**DOI:** 10.1155/2021/8863453

**Published:** 2021-04-24

**Authors:** Tore Bonsaksen, Marte Ørud Lindstad, Carita Håkansson, Petra Wagman, Reinie Cordier

**Affiliations:** ^1^Department of Health and Nursing Sciences, Faculty of Social and Health Sciences, Inland Norway University of Applied Sciences, Elverum, Norway; ^2^Faculty of Health Studies, VID Specialized University, Sandnes, Norway; ^3^Department of Health Sciences, Faculty of Medicine and Health Sciences, Norwegian University of Science and Technology, Gjøvik, Norway; ^4^Division of Occupational and Environmental Medicine, Lund University, Lund, Sweden; ^5^Department of Rehabilitation, Jönköping University, School of Health and Welfare, Jönköping, Sweden; ^6^Department of Social Work, Education and Community Wellbeing, Faculty of Health and Life Sciences, University of Northumbria, Newcastle upon Tyne, UK

## Abstract

**Background:**

Recently, the Occupational Balance Questionnaire developed in Sweden was translated into Norwegian. No studies to date have examined the measurement properties of the Norwegian version of this questionnaire.

**Aim:**

The study is aimed at examining the psychometric properties of the Norwegian version of the Occupational Balance Questionnaire, the OBQ11-N.

**Methods:**

Along with sociodemographic data, 180 occupational therapy students enrolled at two Norwegian universities completed the OBQ11-N as well as one question each related to health and quality of life and some sociodemographic variables. Rasch analysis was employed for examining rating scale functioning, item and person validity, dimensionality, and differential item functioning.

**Results:**

Item categories were ordered, but there were potential gaps in the measurement of the construct. Person reliability was fair, whereas item reliability was low. Point biserial correlations were positive, indicating that all items contributed to the construct. Factor loadings were low for two items, and there were indices of a second underlying dimension and item redundancy. Many people were not aligned with the items, and some items functioned differently across various demographic variables. *Conclusion and Significance*. The OBQ11-N did not function as an adequate measure of occupational balance in a sample of students. Potentially, the detected measurement problems may be solved by adding more relevant items to a larger item pool, from which the best fitting items should be selected.

## 1. Introduction

Occupational therapy practice is based on the belief that occupational performance is linked with the maintenance and restoration of health and life satisfaction [1–3]. However, there is clearly no universal standard concerned with the amount of occupation needed or with how much time should be spent on various kinds of occupations. Thus, the subjective perspective of occupation appears to be important for self-perceived health and life satisfaction. Although objective patterns and subjective perceptions of occupations have been frequently linked in the foregoing literature, Eklund and coworkers [4] suggested treating the two as separate, yet interacting, phenomena. Supporting the emphasis on the subjective experience, Wilcock and coworkers [5] found that participants whose occupational pattern was close to their own ideal balance between different types of occupations had better self-reported health, compared to their counterparts.

To account for the individual's subjective perception of their occupational pattern, the construct of occupational balance has been used. Over the years, different authors have described occupational balance in different ways [6–12]. For example, researchers have emphasized a balance between different aspects of occupation [13], such as balance between challenging versus relaxing occupations, activities considered meaningful to the individual versus meaningful in the sociocultural context, and activities denoting care for oneself versus care for others [14]. Resulting from different conceptualizations, different tools for measuring occupational balance have emerged, such as the Life Balance Inventory [15] and the Occupational Balance Questionnaire (OB-Quest) [16].

Based on a comprehensive literature review and concept analysis, Wagman and coworkers suggested occupational balance to be defined as “the individual's perception of having the right amount of occupations and the right variation between occupations in his/her occupational pattern” (p. 326) [17]. Thus, their definition, which is also used in the current study, addresses both quantitative and qualitative aspects of occupational balance, while exclusively considering the subjective experience and disregarding what the occupations are. Following the concept analysis, the Occupational Balance Questionnaire (OBQ) was proposed as a tool for measuring occupational balance as a unidimensional construct [18]. The study showed that the measure had very high internal consistency (Cronbach′s *α* = 0.94), high test-retest reliability (Spearman′s rho = 0.93), and no indication of floor or ceiling effects (none of the participants received the highest or lowest possible sum score). More recently, a Rasch analysis of the OBQ was conducted based on the responses from two different general population samples [19]. Resulting from the analysis, the revised OBQ11 with 11 items (after two items had been removed) was found to be unidimensional, supporting the construct validity of the measure. Moreover, reducing the number of response categories (each item to be scored 0-3 (lowest to highest)) apparently made it easier for respondents to distinguish between them, there was good person reliability (0.92), and overall, the measure functioned invariably across age and gender groups. Evidence of good psychometric properties has also been found in a Turkish translation of the OBQ11 [20].

A Norwegian version of the measure (OBQ11-N) [21], incorporating the latest developments carried out in Sweden, was recently used in an exploratory study of occupational balance and its association with quality of life in older adults residing in nursing homes in Norway [22]. The study found a significant and positive relationship between higher occupational balance and higher quality of life (*r*_s_ = 0.61) among the male participants, while no such relationship was detected among the female participants. The OBQ11-N had good reliability, as indicated by the internal consistency measure for the eleven items (Cronbach′s *α* = 0.79). However, no other formal psychometric procedures have been conducted with the OBQ11-N. As the study and reporting of psychometric properties of new measures are crucial for their potential uptake in research and practice [23, 24], the current study of the psychometric properties of the OBQ11-N represents a crucial step in the development of a potentially useful measure for occupational therapy research and practice in Norway.

## 2. Study Aim

This study is aimed at examining the psychometric properties of the OBQ11-N, specifically regarding rating scale functioning, person and item fit, dimensionality, and differential item functioning.

## 3. Methods

### 3.1. Participants

Participants were 180 occupational therapy students at two universities in Norway. As the number of eligible students was 227, the overall response rate was 79.3%. The sample size was considered sufficiently large when used with the 11-item OBQ11-N [25]. Occupational therapy education programs are in Norway undergraduate programs of three-year duration, and the students represented all year cohorts. Eighty-three students came from University 1, whereas the remaining 97 students came from University 2. The sample characteristics are displayed in Table 1. The students' mean age was 23.4 years (SD = 3.76); the age range was 19 years to 40 years. The majority (79.3%) were women, and the larger proportion (70.9%) did not have experience from higher education prior to enrolment in the occupational therapy education program.

### 3.2. Measurement

#### 3.2.1. Occupational Balance Questionnaire

The Occupational Balance Questionnaire (OBQ) [18] was developed to measure “the experience of having the right amount of occupations and the right variation between occupations in the occupational pattern” (regardless of what the occupations are). The OBQ is intended to be a generic instrument to evaluate the occupational balance of individuals and groups. Conceptually, the OBQ is based on results from previous research on the experience of occupational balance in different groups [26–28] and a concept analysis of occupational balance [17]. The OBQ focuses on the variation in the occupational pattern including (a) between different types of occupations, between doing things for oneself and for others, between doing things alone, and together with others; (b) the amount of each occupation; and (c) the total amount of occupations (amount of, time for, and number of). In the revised OBQ11-N, which was used in this study, each item has four response options (0 = disagree; 1 = partly agree; 2 = largely agree; 3 = completely agree) [19]. The possible score range was 0-33, with higher scores indicating higher levels of occupational balance. The content of the 11 items is outlined in Table 2.

#### 3.2.2. Health and Quality of Life

Health was measured with one item: “How has your health been during the last week?” Similarly, quality of life (QoL) was measured with one item: “How has your quality of life been during the last week?” These items were taken from the larger assessment battery developed by the European Organisation for Research and Treatment of Cancer Quality of Life Questionnaire (EORTC QLQ-C30) [29] and have been used in clinical research [30] as well as in population surveys [31, 32]. The response format for both items is an 11-point scale anchored by the phrases “very poor” (0) in the lower end and “excellent” (10) in the upper end.

#### 3.2.3. Sociodemographic Variables

Sociodemographic variables included age (years), gender, and highest completed education level.

### 3.3. Statistical Analysis

Rasch analyses were used to evaluate the reliability and validity of the OBQ11-N. The data were analyzed using WINSTEPS version 4.4.7 [33], with the joint maximum likelihood estimation rating scale estimation [34]. The data were analyzed for all 11 Occupational Balance Questionnaire items. In addition to descriptive analyses of occupational balance, health, and quality of life, the following analyses were conducted.

#### 3.3.1. Rating Scale Validity

Examination of the rating scale validity can confirm whether the ordinal response scale for all items stays true to the assumption that higher ratings indicate “more,” and lower ratings indicate “less” of the construct under assessment. In WINSTEPS, rating scale response options are referred to as *categories*. There are three situations in which the partial credit model can be used: (1) items where some responses may be more correct than others, (2) items that can be broken down into component tasks, and (3) items where increments in the quality of performance are rated [35]. None of these situations apply to the OBQ11 scale structure, and all OBQ11 items have the same scale structure. As such, a Rating Scale Model (RSM) was used. In alignment with the OBQ11 response options, the categories are numbered from 0 to 3.

To determine if the rating response scales were being used in the expected manner, category response data was examined for even distribution or category disorder. Poorly defined categories or the inclusion of items do not measure the construct result in nonuniformity/disordering. Ordered categories are indicated by average measure scores (frequency of use) that increase monotonically as the category increased. Mean squares (MnSq) outside 0.7-1.4 indicate category misfit and disordering, and the collapsing of the misfitting category with an adjacent category should be considered [36].

The point at which there is equal probability of a response in either of two adjacent categories being selected, known as step calibrations or Andrich thresholds, was determined to assess step disordering. Andrich thresholds reflect the distance between categories and should progress monotonically, showing neither overlap between categories nor too large a gap between categories. Step disordering indicates that the category defines a narrow section of the variable but does not imply that the category definitions are out of sequence. The average measure distinct categories are indicated by an increase of at least 1.0 logit on a 5-category scale. An increase of >5.0 logits, however, is indicative of gaps in the variable [37].

#### 3.3.2. Person and Item Fit Statistics

Construct validity was assessed using fit statistics to identify misfitting items and the pattern of responses for each person. Fit statistics are reported as log odd units (logits) and indicate whether the items contribute to the one construct (i.e., occupational balance) and the degree to which a person's responses are reliable. Unstandardized MnSq or *Z*-standard (*Z*-STD) scores can be used to describe infit and outfit MnSq values which should be close to 1.0 with an acceptable range of 0.7-1.4 [38]. The outfit *Z*-STD values are expected to be 0, and any value that exceeds ±2 is interpreted as less than the expected fit to the model [38]. Model underfit degrades the model and requires further investigation to determine the reason for the underfit. Model overfit, on the other hand, does not always degrade the model but still can lead to the misinterpretation that the model worked better than expected [38].

The internal consistency of the measure is evaluated through the person reliability, which is equivalent to the traditional Cronbach's alpha. Low person reliability values (<0.8) indicate having too few items or a narrow range of person measures (i.e., not having enough persons with more extreme abilities, both high and low).

If outlying measures are accidental, people are classified using person separation. However, if the outlying measures represent true performance, people are classified using the person separation index (PSI) (4∗person separation + 1/3). To distinguish high performers from low performers, the person separation index determines whether the test separates the sample into distinct levels. Low person separation is indicative that the measure is not sensitive enough to separate low and high performers. Reliability of 0.5, 0.8, and 0.9, respectively, indicates separation into only one or two levels, 2 to 3 levels, and 3 to 4 levels [36]. A PSI of 3 is required (the minimum level to attain a reliability of 0.9) to consistently identify three levels of performance. Item hierarchy with <3 levels (high, medium, and low) is verified by item reliability. If item reliability < 0.9, the sample is too small to confirm the construct validity (item difficulty) of the measure.

#### 3.3.3. Dimensionality of the Scale

Dimensionality can be assessed by the following means: (a) using negative point-biserial correlations to identify any potentially problematic items, (b) identifying misfitting persons or items using Rasch fit indicators, and (c) performing Rasch factor analysis using principal component analysis (PCA) of the standardized residuals [39]. The number of principal components is checked using PCA of residuals to confirm that there are no second or further dimensions after the intended or Rasch dimension is removed. No second dimension is indicated if the residuals for pairs of items are uncorrelated and normally distributed. The following recommended criteria are used to determine if further dimensions in the residuals are present: (a) the Rasch factor uses a cut-off of >60% of the explained variance, (b) on first contrast the eigenvalue of <3 (equivalent to three items), and (c) the first contrast of <10% of explained variance [36].

The person-item dimensionality map using a logit scale schematically represents the distributions of the person abilities and item difficulties [33]. In this paper, person ability refers to a self-reported level of occupational balance. “Difficult” items on the OBQ11 would attempt to capture aspects of occupational balance that occur with such infrequency that very few assessors will give a high rating to these items, whereas “easy” items might refer to aspects of occupational balance that occur regularly and will receive high assessors' ratings [33]. If two or more items represent similar difficulty, these items occupy the same location on the logit scale. Locations on the logit scale where persons are represented with no corresponding item identify gaps in the item difficulty continuum. The person measure score is another indicator of overall distribution. A person's mean measure score location on the person-item map, lower than the centralized item mean score of 50, indicates people in the sample were more able than the level of difficulty of the items. If the mean person location is higher (above 50), then the people in the sample were less able than the mean item difficulty.

#### 3.3.4. Differential Item Analysis

To examine whether the scale items were used in the same way by all groups, a differential item functioning (DIF) analysis was performed. DIF occurs when a characteristic other than the occupational balance difficulty being assessed influences their rating on an item [38]. For DIF analysis, the distribution of the included variables was considered. The variables were categorized based on the principle of ensuring comparable number of participants per category or, if not possible, by using the predetermined categories used in the demographic information section of the questionnaire. These principles were applied to the following variables: age (19-21 years vs. 22-23 years vs. 24-29 years vs. 30-40 years), level of education (completed high school vs. completing 3 years or more of previous higher education), health (health scores 0-4 vs. health scores 5-6 vs. health scores 7-8 vs. health scores 9-10), gender (male vs. female), quality of life (quality of life scores 0-4 vs. quality of life scores 5-6 vs. quality of life scores 7-8 vs. quality of life scores 9-10), and level of occupational balance (occupational balance scores 0-15 vs. occupational balance scores 16-19 vs. occupational balance scores 20-33). These variables were selected for inclusion in the DIF analysis as they might explain differences between groups in terms of their occupational balance. For example, younger students may have recently established themselves on their own and may be relatively new to the study situation altogether, whereas this would not be the case for older students. Similarly, poor health might indicate activity and/or participation restrictions, and health differences may therefore contribute to explain differences in occupational balance. DIF contrast is inspected when comparing groups and refers to the difference in difficulty of the item between groups. When testing the hypothesis “this item has the same difficulty for two groups,” DIF is noticeable when the DIF contrast is at least 0.5 logits with a *p* value < 0.05. In determining DIF when comparing more than two groups (e.g., age groups) with the hypothesis “this item has no overall DIF across all groups,” the chi-square statistic and *p* value < 0.05 are used [36]. WINSTEPS implements two DIF methods: first is the widely used Mantel-Haenszel and Mantel methods which are (log-)odds estimators of DIF size and significance from crosstabs of observations of the two groups and the second is a logit difference (logistic regression) method, which estimates the difference between the Rasch item difficulties for the two groups, holding everything else constant. For the DIF analysis conducted in this analysis, we used the Mantel-Haenszel test for dichotomous variables and the Mantel test for polytomous variables as these methods are generally considered most authoritative.

### 3.4. Ethics and Procedure

The Norwegian Centre for Research Data approved the study (reference 713089). Data were collected in February-March 2019 from occupational therapy students at two different universities. The project representatives at each of the universities informed the students about the purpose and procedures of the study on the relevant digital learning platforms, as well as verbally before collecting the data. The students completed the questionnaire by paper and pencil as part of a classroom session. No direct person-identifying information was collected. All students were informed that participation in the study was voluntary and that completing the questionnaire was considered as informed consent to participate.

## 4. Results

Calculating occupational balance as the sum score of the 11 items, the mean level of occupational balance in the sample was 18.0 (SD = 6.1; Md = 18.0; range: 2-33). Mean overall health was 7.1 (SD = 2.0), whereas mean quality of life was 6.8 (SD = 2.1). Next, the study examined the psychometric properties of the OBQ11-N, specifically regarding rating scale functioning, person and item fit, dimensionality, and differential item functioning.

### 4.1. Rating Scale Validity

**Figure 1:**
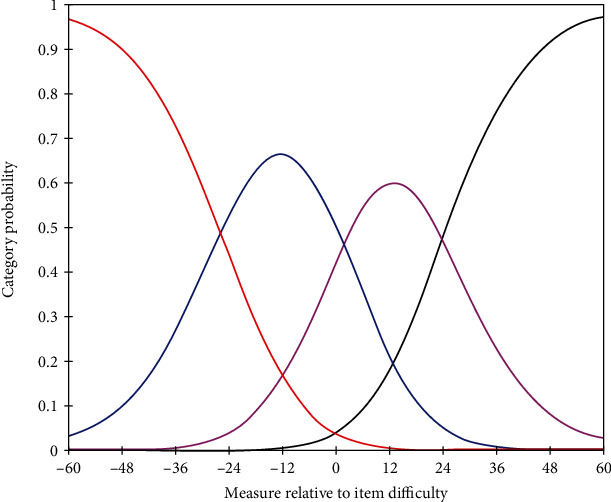
Rating scale validity.

The OBQ11-N uses a 4-point (0-3) rating scale to rate the person's subjective experience of occupational balance. For the overall instrument, the probability of a category being observed was examined. Category “0” was rarely used (8% of ratings). The average measure scores increased monotonically, and the fit statistics were all in the acceptable range (MnSq > 0.7 and <1.4) resulting in four distinct, ordered categories (see Table 3; Figure 1). When examining the Andrich thresholds that reflect the relative frequency of use of the categories, they were not disordered, but all categories advanced by >5 logits (range -25.86 to 24.06 logits), indicating potential gaps in the measurement of the variable (i.e., in the category labels) such that adding more response options would be recommended.

### 4.2. Person and Item Fit Statistics

The summary infit and outfit statistics for item and person ability for the 11-item scale showed good fit to the model with a low item reliability estimate (0.85), which is below the required level of 0.90 to confirm the hierarchy of the scale items, and fair person reliability (0.85). The PSI of 3.64 was only marginally above the minimum of 3 required to separate people into distinct groups based on their occupational balance scores (see Table 4).

We then examined item misfit for all individual 11 items (see Table 5). We examined infit and outfit scores for contradictions and found a similar number reported misfitting infit and outfitting *Z*-STD, and there were no contradictions in the direction of change. Underfit (MnSq > 1.4; *Z* − STD > 2) is the biggest threat to the measure because it can degrade the model as it occurs because of too much variation in the responses [38]. Underfit of both infit and outfit scores was observed for item 11. More misfit was evident on infit and outfit *Z*-STD scores than MnSq with the *Z*-STD infit and outfit also underfitting for items 1, 5, and 11. Overfit (MnSq < 0.7; *Z* − STD < −2) of the MnSq and *Z*-STD infit and outfit scores for items 9 and 10 were observed, as well as *Z*-STD overfit for item 2 and outfit overfit for item 6. Point biserial correlations were examined and all found to be in a positive direction, indicating all items contribute to the overall construct. Factor loadings were low for items 4 and 6 (0.05 and 0.01, respectively).

### 4.3. Dimensionality

The dimensionality of the overall scale with all 11 items was examined using principal components analysis (PCA) of the residuals (see Online Supplement 1: dimensionality of the scale). The Rasch dimension explained 44.6%. However, of the 44.6% explained variance, the person measures (27.4%) explained almost twice the variance explained by item measures (17.2%). The total raw unexplained variance (55.4%) had an eigenvalue of 11, resulting in the eigenvalue of first contrast being 1.94. The PCA of residuals divided the items into two groups (items 2, 4, 5, 10, and 11 and items 1, 3, 6, 7, 8, and 9, respectively).

As displayed in Online Supplement 1, a second dimension was considered against the criteria. While the PCA revealed that the explained variance (44.6%) was below the 60% cut-off to indicate a second dimension, the eigenvalue (1.94) was below the required eigenvalue of 3 on the first contrast and the unexplained variance (9.8%) was less than the required 10% on the first contrast. This together with a deattenuated correlation of 0.69 in the first contrast on item clusters 1 and 3 suggests there may be the emergence of a second dimension. Further support for a second dimension is low factor loadings for items 4 and 6.

**Figure 2:**
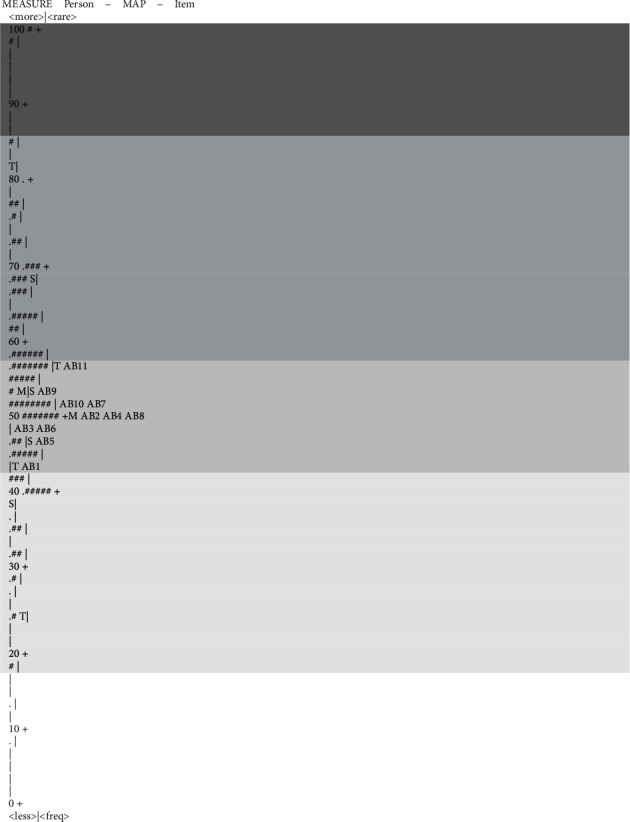
Person-item map. Note: each “#” is 2, each “.” is 1.

The person-item dimensionality map (as presented in Figure 2) shows that many people were not aligned with the items. Item redundancy indicated by items occupying the same location on the logit scale was present for items 2, 4, and 8, items 7 and 10, and items 3 and 6. The person-item dimensionality map also shows the lack of easy and difficult items, with no items against a large number of persons at both the bottom and top ends of the map. This, together with the large number of redundant items, is indicative for the need to generate more items to fully capture the construct of occupational balance.

### 4.4. Differential Item Analysis

The DIF analysis enabled examination of potential contrasting item-by-item profiles associated with (a) age, (b) gender, (c) level of education, (d) health, (e) quality of life, and (f) level of occupational balance. The summary of the DIF analysis for all 11 items is presented in Online Supplement 2: summary DIF analysis. Significantly, different responses on four of the 11 items were based on participant category for age (item 1), gender (item 7), education (item 5), QoL (items 3 and 5), and occupational balance (items 1 and 5). These results indicated that there is some item bias, which would vary the hierarchy of items across samples.

The summary of the DIF analysis for the 11 items (presented in Online Supplement 2) revealed that participant categories QoL (groups 1 vs. 2 vs. 3 vs. 4) and occupational balance (groups 1 vs. 2 vs. 3) were the major factors in how items were used. In terms of QoL, DIF on the identified items indicated that students in group 3 (group with the second highest ratings of QoL) scored higher than expected on item 3 and students in group 4 (group with the highest ratings of QoL) scored higher than expected on item 5. In terms of occupational balance, students in group 1 (group with the lowest level of occupational balance) scored lower than expected on both items 1 and 5. That is, students with higher ratings in QoL (groups 3 and 4) found items 1 and 5, respectively, easier than expected. Conversely, students with the lowest ratings in terms of occupational balance (group 1) found items 1 and 5 more difficult than expected. In relation to age, students who were older (30-40 years of age) found item 1 easier than expected. In terms of gender, males found item 7 more difficult than expected, and for education, students who completed a previous degree found item 5 more difficult than expected.

## 5. Discussion

While occupational balance is an important construct in the occupational therapy profession, researchers have conceptualized and measured it in different ways. In Sweden, one consistent line of research has led to the development and validation of the OBQ as a measure of occupational balance [18, 19]. A Turkish translation of the OBQ11 was found to possess good measurement properties [20], and the current study is aimed at examining the psychometric properties of the Norwegian instrument version. Overall, we found that the OBQ11-N did not function adequately as a measure of occupational balance in a sample of occupational therapy students, and the various reasons are discussed below together with implications and suggestions.

### 5.1. Rating Scale Validity

The OBQ11-N items had ordered functioning, but there were potential gaps in the measurement of the variable. The original OBQ comprised six response categories, which was reduced to four response categories in the OBQ11. The results indicate that five response options might be a better balance. The lowest rating category (i.e., category 0: disagreeing with the content of the OBQ items) was rarely used (8% of total sample). One should keep in mind that the sample was comprised of young and relatively healthy students enrolled in a higher education program, their age and resourcefulness possibly indicating a potential for rating the OBQ11-N items higher. In comparison, a study of persons with stroke found that the participants were more inclined to use the lowest ratings with the OBQ items (up to 19%) [40]. However, a recent study of nursing home residents in Norway found relatively high occupational balance scores among the participants, despite their old age and chronic health problems [22]. Only one participant (2.2%) scored zero. Thus, the relationship between age and health and occupational balance does not seem to be linear and is likely influenced by a range of confounding variables. An emphasis on occupational balance or imbalance as a *subjective experience*, as defined by Wilcock and Hocking [2, 5] and the occupational balance definition underpinning the OBQ measure [17], may explain variations in occupational balance scores.

### 5.2. Person and Item Fit Statistics

In a recent validation study conducted in Sweden [19], the OBQ items followed a logical order from easier (e.g., “time for doing things wanted”) to more difficult items (e.g., “balance between work, home, family, leisure, rest and sleep”). In the current study, however, we were unable to confirm the hierarchy of the scale items as the item reliability estimate (0.85) was found to be below the required level. Thus, when used with the current sample, the logical order of items (from easier to more difficult) was absent, rendering it difficult to compare persons' occupational balance by comparing their scores on individual items. Moreover, person reliability was marginal (0.86), suggesting the measure has too few items and/or the sample did not have enough persons with very high or low occupational balance “ability.” This finding is further supported by a marginal PSI score, indicating the measure has difficulty in separating people into distinct occupational balance strata. This decreases the usefulness of the OBQ11-N in clinical practice settings, as the ability to classify persons into groups of high, medium, or low occupational balance would be reduced. In comparison, Håkansson and coworkers [19] found a reliability measure of 0.92, allowing the separation of persons into distinct groups based on their scores on the OBQ11. It seems likely that the use of a narrowly composed sample, such as the young occupational therapy students in the current study, would display less variation in their occupational balance scores, compared to the variation shown in general population samples, such as those used in the study from Sweden [19].

### 5.3. Individual Item Level

An underfit of both infit and outfit scores was observed for item 11. The *Z*-STD infit and outfit were also underfitting for items 1, 5, and 11 and overfit for items 9 and 10. There was a *Z*-STD overfit for item 2 and an outfit overfit for item 6. Taken together, the results indicate that several items showed a poor fit to the Rasch model [38]. However, point biserial correlations were all in the positive direction, indicating that all items contributed to the latent construct (occupational balance). Factor loadings were low for items 4 and 6, indicating the possibility of a second latent dimension. Taken together, the misfitting items appear to over- or underdiscriminate relative to the summary discrimination of all items on the measure [19, 41]. Thus, the results point to the need to explore a larger pool of items that could contribute to an improvement of the measure.

### 5.4. Dimensionality

The PCA revealed that the explained variance was below the cut-off of the applicable parameter, which indicates one main latent dimension. In addition, the eigenvalue of the first contrast was below the required level to suggest a second dimension, and the unexplained variance was less than the required threshold on the first contrast [36]. However, one should also consider the low factor loadings for some of the items (items 4 and 6) and that most of the explained variance was related to persons and not items. These findings, together with a low deattenuated correlation in the first contrast, suggest the possible emergence of a second dimension latent in the measure. However, detecting a pattern across misfitting items and items with low factor loadings to identify the nature of the potential second dimension appears to be difficult. None of the previous psychometric studies of the OBQ11 in more diverse samples has indicated a second latent dimension [19, 20], suggesting that the current study's indication of a second latent dimension should be investigated in a larger and more diverse context.

Moreover, the person-item dimensionality map (Figure 2) shows that many people were not aligned with the items. Item redundancy was present for items 2, 4, and 8, for items 7 and 10, and for items 3 and 6. Essentially, while the participants showed a certain distribution across the ruler (fair person fit), the person-item dimensionality map shows the lack of easy and difficult items—all items had about the same level of difficulty. At least for the current sample, the low item fit and the large number of redundant items indicate a need to generate more items to capture more fully the construct of occupational balance.

### 5.5. Differential Item Analysis

Significantly different responses between groups of participants were found for item 1 (difference between age group and occupational balance groups), for item 3 (difference between quality of life groups), for item 5 (difference between education groups, quality of life groups, and occupational balance groups), and for item 7 (difference between genders). In view of these results, there is indication of some item bias, which would vary the hierarchy of items across samples.

Two of the significant group differences were concerned with different levels of quality of life. These results appear to mirror the results of previous studies, in which occupational balance has been shown to be associated with quality of life or life satisfaction [42, 43]. Thus, it seems logical that different levels of quality of life would influence a person's rating of some of the OBQ11 items. Despite the relatively homogeneous sample, an age-based difference was found for item 1, and different levels of overall occupational balance influenced different scores on two items. These findings have, so far, no comparison in the previous literature. Thus, group differences on OBQ11 items should continue to be investigated in future studies, as consistent group differences will reduce the scope of the population for which OBQ11 data can be considered valid.

### 5.6. Study Limitations and Suggestions for Future Research

The sample used in this study was very homogeneous, largely consisting of young and resourceful students in relatively good health. Thus, the results have limited external validity, in comparison to studies using samples drawn from the general population. For example, the sample employed by Håkansson and coworkers [19] ranged between 20 and 89 years of age, while the sample in the current study ranged 19-40 years, with 94% being 30 years or younger. Moreover, the sample was comprised of occupational therapy students, thus students training for a profession for which the construct of occupational balance is of particular importance and value. The students also shared experiences of having periods with exams, often accompanied by stress and potentially less sleep and less diversity in occupations. Thus, the highly specific sample composition in the current study may contribute to explain the differences in relation to previous research. Consequently, future studies may continue to explore the OBQ11-N in samples better representing the general Norwegian population. Otherwise, the size of the sample, the data collection procedures, and the sophisticated analysis employed all suggest high internal validity of the results.

## 6. Conclusion

While previous studies have shown good psychometric properties for the OBQ measure, this was not replicated in the current study of the Norwegian version of the OBQ11. In particular, the problems demonstrated for the items in the measure indicate it might be wise to return to the development stage in designing a measure of occupational balance to be used in the Norwegian context. Preferably, generating a larger pool of items from which to choose might contribute to solve some of the problems demonstrated in this study. However, the study is limited in its use of a highly specific sample, and future studies should strive to employ samples of more diversity, preferably general population samples.

## Figures and Tables

**Table 1:** Participant demographics.

Participants	*N* = 179^∗^	%
Age range		
19–21 years	57	31.8
22–23 years	62	34.6
24–29 years	45	25.1
30–40 years	15	8.4
Gender		
Male	34	20.7
Female	145	79.3
Level of education		
Completed high school	127	71.0
Previous higher education experience	52	29.0
Health (0-10 scale)		
Group 1 (health score 0-4)	23	12.9
Group 2 (health score 5-6)	41	22.9
Group 3 (health score 7-8)	70	39.1
Group 4 (health score 9-10)	45	25.1
Quality of life (0-10 scale)		
Group 1 (quality of life score 0-4)	31	17.3
Group 2 (quality of life score 5-6)	48	26.8
Group 3 (quality of life score 7-8)	61	34.1
Group 4 (quality of life score 9-10)	39	21.8
Occupational balance (0-33 scale)		
Group 1 (occupational balance score 0-15)	57	31.7
Group 2 (occupational balance score 16-19)	57	31.7
Group 3 (occupational balance score 20-33)	66	36.6

Note: ^∗^demographic data were missing for one student.

**Table 2:** Content of items of the OBQ11-N.

Item #	Item content
1	Having just enough to do during a regular week
2	Balance between doing things for others and for oneself
3	Time for doing things wanted
4	Balance between work, home, family, leisure, rest, and sleep
5	Have sufficient time for doing obligatory occupations
6	Balance between physical, social, mental, and restful occupations
7	Satisfaction with how time is spent in everyday life
8	Satisfaction with the number of activities during a regular week
9	Balance between obligatory and voluntary occupations
10	Balance between energy-giving and energy-taking activities
11	Satisfaction with time spent in rest, recovery, and sleep

Note: all items are scored 0-3 (lower level-higher level).

**Table 3:** Rating scale validity of the OBQ11-N.

Response category	*N*	%	Average measures	Infit MnSq	Outfit MnSq	Andrich thresholds
0	149	8	-20.07	0.86	0.87	None
1	727	37	-2.45	1.02	1.01	-25.86
2	794	40	8.17	0.96	0.96	1.80
3	303	15	19.42	1.10	1.12	24.06

Note: missing data = 7; 0.004%; observed averages are the means of measures in category. It is not a parameter estimate.

**Table 4:** Item and person summary statistics.

Items	Item/person	Reliability	Separation	PSI^∗^	Mean measure	Model SE	Infit		Outfit	
							MnSq	*Z*-STD	MnSq	*Z*-STD
All 11 items	Item	0.85	2.42	—	50.00	1.23	1.00	-0.29	1.00	-0.28
	Person	0.86	2.48	3.64	53.67	5.15	1.00	-0.06	1.00	-0.06

Note: Cronbach's alpha (KR-20) person raw score “test” reliability = 0.88; SEM = 2.11; ^∗^person separation index (PSI)/strata = (4∗person separation + 1/3).

**Table 5:** Individual item fit statistics and principal component analysis for all 11 items combined.

Items	Mean measure	SE	Infit		Outfit		Factor loading	Point biserial correlations
			MnSq	*Z*-STD	MnSq	*Z*-STD		
1	43.45	1.23	1.27	2.54	1.28	2.51	-0.24	0.49
2	49.41	1.22	0.78	-2.30	0.78	-2.30	0.19	0.68
3	47.75	1.23	1.01	0.12	1.02	0.23	-0.16	0.63
4	49.56	1.22	0.99	-0.06	0.98	-0.20	0.05	0.70
5	46.34	1.23	1.31	2.85	1.33	2.88	0.51	0.53
6	48.82	1.22	0.82	-1.92	0.80	-2.09	-0.01	0.73
7	52.39	1.22	0.82	-1.87	0.83	-1.77	-0.69	0.74
8	50.45	1.22	1.16	1.54	1.19	1.81	-0.54	0.65
9	53.44	1.22	0.64	-4.06	0.64	-3.99	-0.41	0.76
10	51.77	1.23	0.54	-5.37	0.54	-5.39	0.34	0.81
11	56.61	1.23	1.66	5.37	1.64	5.20	0.71	0.60

Note: 37 (20.6%) persons had poor infit underfit (MnSq > 1.4); 57 (31.7%) persons had poor infit overfit (MnSq < 0.7).

## Data Availability

The data used to support the conclusions of this study can be obtained from the corresponding author on reasonable request.

## Supplementary Materials

Supplementary 1 Online Supplement 1: dimensionality of the scale.

Supplementary 2 Online Supplement 2: summary DIF analysis.
