# Supplementary material for: Rasch Analysis of the Norwegian Version of the Occupational Balance Questionnaire in a Sample of Occupational Therapy Students
Source: Occup Ther Int. 2021 Apr 24;2021:8863453. doi: 10.1155/2021/8863453 (PMC8088501; doi:10.1155/2021/8863453)
Supplement: Supplementary 1 — Online Supplement 1: dimensionality of the scale. [file 8863453.f1.docx]

Online Supplement 1. Dimensionality of the scale

| Variance | Eigenvalue | Observed (%) | Expected (%) |
| --- | --- | --- | --- |
| Total raw variance in observations | 19.85 | 100.0% | 100.0% |
| Raw variance explained by measures | 8.85 | 44.6% | 44.6% |
| Raw variance explained by persons | 5.44 | 27.4% | 27.4% |
| Raw Variance explained by items | 3.41 | 17.2% | 17.2% |
| Raw unexplained variance (total) | 11.00 | 55.4% | 55.4% |
| Unexplained variance in 1st contrast | 1.94 | 9.8% | 17.7% |
| Unexplained variance in 2nd contrast | 1.60 | 8.1% | 14.5% |
| Unexplained variance in 3rd contrast | 1.28 | 6.5% | 11.7% |
| Unexplained variance in 4th contrast | 1.18 | 6.0% | 10.7% |
| Unexplained variance in 5th contrast | 1.05 | 5.3% | 9.5% |
