# Supplementary material for: Rasch Analysis of the Norwegian Version of the Occupational Balance Questionnaire in a Sample of Occupational Therapy Students
Source: Occup Ther Int. 2021 Apr 24;2021:8863453. doi: 10.1155/2021/8863453 (PMC8088501; doi:10.1155/2021/8863453)
Supplement: Supplementary 2 — Online Supplement 2: summary DIF analysis. [file 8863453.f2.docx]

Online Supplement 2. Summary DIF analysis

| Item | Age | | | Gender | | | Education | | |
| --- | --- | --- | --- | --- | --- | --- | --- | --- | --- |
|  | Summary DIF  Chi-squared | Prob. | DIF contrast  (effect size)^⸸^ | Mantel–  Haenszel Prob. | Prob. | DIF contrast  (effect size) ^ꞎ^ | Mantel–  Haenszel Prob | Prob. | DIF contrast  (effect size)^ |
| 1 | 11.3285 | .0230* | -4.51* | .6393 | .4240 | -6.37 | .0365 | .8485 | -1.67 |
| 2 | 1.8809 | .7574 | 1.24 | 1.2806 | .2578 | 1.74 | .0987 | .7533 | -3.16 |
| 3 | 1.2129 | .8759 | -0.28 | 1.6575 | .1979 | -4.66 | 1.2203 | .2693 | -1.63 |
| 4 | 2.2403 | .6913 | -1.69 | .0392 | .8431 | 3.63 | .9394 | .3324 | -2.22 |
| 5 | 5.2717 | .2600 | -2.80 | 1.3444 | .2463 | 3.41 | 6.3494 | .0117* | 6.30* |
| 6 | 2.3915 | .6638 | -0.21 | .0012 | .9726 | .00 | .4965 | .4810 | -.63 |
| 7 | 3.0065 | .5563 | 3.63 | 5.5199 | .0188* | -7.14* | .0451 | .8317 | .87 |
| 8 | 3.5407 | .4712 | 4.50 | .1878 | .6647 | .26 | .0025 | .9603 | .92 |
| 9 | 6.3963 | .1710 | 2.08 | 2.6312 | .1048 | 4.05 | 3.4088 | .0648 | 2.55 |
| 10 | 1.4640 | .8329 | 2.08 | .0069 | .9338 | -2.65 | .5972 | .4397 | 1.46 |
| 11 | 2.6950 | .6097 | 2.10 | 1.1522 | .2831 | 7.10 | 1.0301 | .3101 | -2.85 |

Online Supplement 2. Summary DIF analysis (Cont.)

| Item | Health | | | QoL | | | Occupational Balance | | |
| --- | --- | --- | --- | --- | --- | --- | --- | --- | --- |
|  | Summary DIF  Chi-squared | Prob. | DIF contrast  (effect size)^#^ | Summary DIF  Chi-squared | Prob. | DIF contrast  (effect size)^Ɥ^ | Summary DIF  Chi-squared | Prob. | DIF contrast  (effect size)^Ꞷ^ |
| 1 | 3.9296 | .4150 | -1.18 | 5.9931 | .1992 | -1.95 | 15.3716 | .0004* | -9.29* |
| 2 | 1.9685 | .7413 | -3.20 | 1.1727 | .8825 | .22 | 1.2213 | .5400 | -.70 |
| 3 | 4.8273 | .3049 | -4.48 | 12.5456 | .0137* | -5.50* | 3.3959 | .1803 | -5.28 |
| 4 | 1.6460 | .8004 | -0.83 | 3.9694 | .4096 | 4.21 | 4.4716 | .1049 | 5.30 |
| 5 | 2.6467 | .6182 | -2.22 | 13.8117 | .0079* | -5.11* | 8.3644 | .0149* | -7.30* |
| 6 | 2.4932 | .6455 | 2.89 | 4.8271 | .3049 | 4.75 | 2.8907 | .2324 | 4.59 |
| 7 | .8546 | .9310 | 2.11 | 2.7419 | .6015 | 1.88 | 3.1152 | .2076 | 3.99 |
| 8 | 5.6717 | .2245 | 1.91 | 2.4923 | .6456 | 2.99 | .0579 | .9731 | -.42 |
| 9 | 6.1377 | .1886 | 10.37 | .7902 | .9398 | 0.72 | .5495 | .7593 | 1.25 |
| 10 | 3.3692 | .4975 | -0.38 | 1.3479 | .8531 | 1.46 | 2.7854 | .2451 | 4.30 |
| 11 | 4.3385 | .3615 | -5.01 | 5.8773 | .2080 | -3.04 | 2.6482 | .2627 | 5.23 |

*Note*. Chi-square statistic used for more than two categories; Mantel-Haenszel test are used for dichotomous; ^⸸^Age: 19-21 years vs. 22-23 years vs. 24-29 years vs. 30-40 years (19-21 years reference group); Gender: ^ꞎ^Male vs. Female (Male reference group); ^Education: completed school vs. completed previous degree (completed school reference group); ^#^Health: group 1 vs. group 2 vs. group 3 vs. group 4 (group 1 reference group); ^Ɥ^Quality of Life: group 1 vs. group 2 vs. group 3 (group 1 reference group); ^Ꞷ^Occupational balance: group 1 vs. 2 vs. group 3 (group 1 reference group); *Denotes items with *p* < 0.05 and Effect size (DIF contrast) > 0.5.
